# Sex differences in adult craniofacial parameters

**DOI:** 10.1007/s00276-015-1477-9

**Published:** 2015-05-03

**Authors:** Suat Avci, Tarkan Ergun, Erdinc Aydin, Leyla Kansu

**Affiliations:** Department of Otolaryngology-Head and Neck Surgery, Baskent University, Saray Mah. Yunusemre Cad. No. 1, Alanya, 07400 Antalya, Turkey; Department of Radiology, Baskent University, Ankara, Turkey

**Keywords:** Anatomy, Skeleton, Head, Chronic otitis media, Computed tomography

## Abstract

**Purpose:**

To compare normal male and female craniofacial parameters in adults and evaluate associations of sex and intercochlear distance with other craniofacial parameters.

**Methods:**

In 60 normal adults (30 men and 30 women) who had no otitis media, craniofacial parameters were measured retrospectively on two-dimensional reformatted computed tomography scans.

**Results:**

Compared with women, men had significantly greater mean osseous auditory tube length, cartilaginous auditory tube length, mastoid length, intercochlear distance, sella to posterior nasal spine distance, sella to basion distance, and nasopharynx sagittal area. The intercochlear distance was significantly correlated with mastoid depth, midpoint of the pharyngeal opening distance, sella to nasion distance, and nasopharynx sagittal area and inversely with angle of the auditory tube. Most men and women had Körner septum present, and mean thickness of Körner septum was significantly greater in men than women.

**Conclusions:**

Some craniofacial parameters, especially vertical parameters, differ with sex. These differences begin in childhood and continue in adulthood. Sex must be considered when planning a craniofacial morphologic study, and results of a craniofacial morphologic study should be evaluated with caution when there is no sex matching of the patient and control groups.

## Introduction

In infants and children, synchronized growth of the craniofacial skeleton is associated with development of the auditory tube system. Abnormal growth or synchronization during development may adversely affect the anatomy and function of the auditory tube system and predispose to otitis media [3, 10]. Most cases of otitis media with effusion resolve spontaneously by age of 10 years, but chronic otitis media is a common problem worldwide [12]. It is unknown how or why otitis media may become chronic, but genetic and environmental factors may contribute to disease progression [19].

Patients who have chronic otitis media may differ from control groups in nasopharyngeal size [12], facial morphology [4, 5, 10, 13], length and angle of cranial base components [5, 10], the bony portion of the auditory tube [12, 22], projections of the auditory tube and associated musculature onto the parasagittal plane [10], mastoid size and degree of pneumatization, jugular bulbus height, and presence and size of Körner septum. These relations have been evaluated in cadaver specimens with lateral cephalometric radiographs, computed tomography (CT), and magnetic resonance imaging. However, it is difficult to synthesize these results because of differences in study design and technique [3]. Cephalometry may not image the auditory tube system directly, and the proposed effects of craniofacial development on the auditory tube system are inferred [3]. In addition, studies vary in patient age, and craniofacial patterns and auditory tube-middle ear system morphology may vary with age [3].

In a previous study, two-dimensional reformatted CT images were used for cephalometric assessment, and when sex, age and race are considered there were no differences in most craniofacial parameters between adults who had chronic otitis media and healthy subjects, except small mastoid size (mastoid depth and length) correlated with chronic otitis media [1].

However, some previous craniofacial morphologic studies did not evaluate the effect of sex on craniofacial parameters. Compared with women, men have more massive muscles and body organs, their lungs are larger, and they need a larger airway beginning with the nose and nasopharynx; these differences in size and configuration of the nose may cause collateral differences in other topographic structures [6]. In addition, morphologic measurements in normal subjects may be useful in the study of patients who have chronic otitis media.

The purpose of the present study was to evaluate and compare craniofacial parameters in normal male and female Turkish adults using two-dimensional reformatted CT scanning, and to evaluate the effect of sex and breadth of the cranial base, measured by the intercochlear distance (IcD), in craniofacial morphologic studies. These data may provide additional information to verify more realistically the association between otitis media and craniofacial morphology.

## Materials and methods

### Subjects

This retrospective study was performed with two-dimensional reformatted CT scans from 60 adults who had healthy ears. There were 30 men (age: mean 37 years; range 19–74 years) and 30 women (age: mean 39 years; range 25–72 years). The subjects were selected from patients who had been referred for a CT scan for evaluation of a space-occupying lesion, trauma, or malformation of the temporal bone. Patients who had CT abnormalities of the internal auditory canal or temporal bone were excluded from the study. Criteria for a normal temporal CT scan included absence of bony destruction or sclerosis, absence of fluid or mass in all temporal bone air spaces, and presence of normal air cells with a sharp, well-defined mucoperiosteal margin and no septal thickening.

### Imaging

The CT scans (Siemens Hi-Speed CT scanner, Siemens, Erlangen, Germany) were performed with scan parameters 120 kV, 300 mA, 1-mm slice thickness, zero interslice gap, and 512 × 512 matrix. The axial scans were parallel to the orbitomeatal line. The image data were transferred to a workstation (GE Advantage, Windows version 4.2, GE Medical Systems, Wilmington, MA, USA). An experienced radiologist and otolaryngologist worked together to reformat each CT scan for accurate landmark identification.

**Fig. 1:**
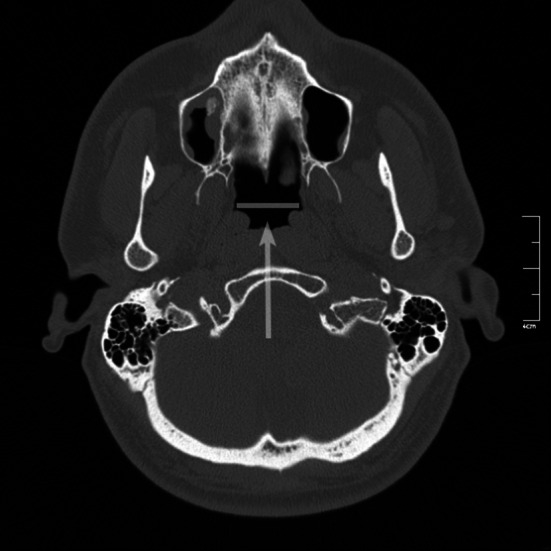
Craniofacial computed tomography scan. Pharyngeal openings of the auditory tube were determined on the axial plane. The *cursor* (*arrow*) was placed in the middle of a line joining the openings of the auditory tube

Axial images were transformed to oblique reformatted images to measure auditory tube length. The pharyngeal openings of the auditory tube were determined on the axial plane, and a cursor was placed in the middle of a line joining the openings (Fig. 1).

### Auditory tube parameters

**Fig. 2:**
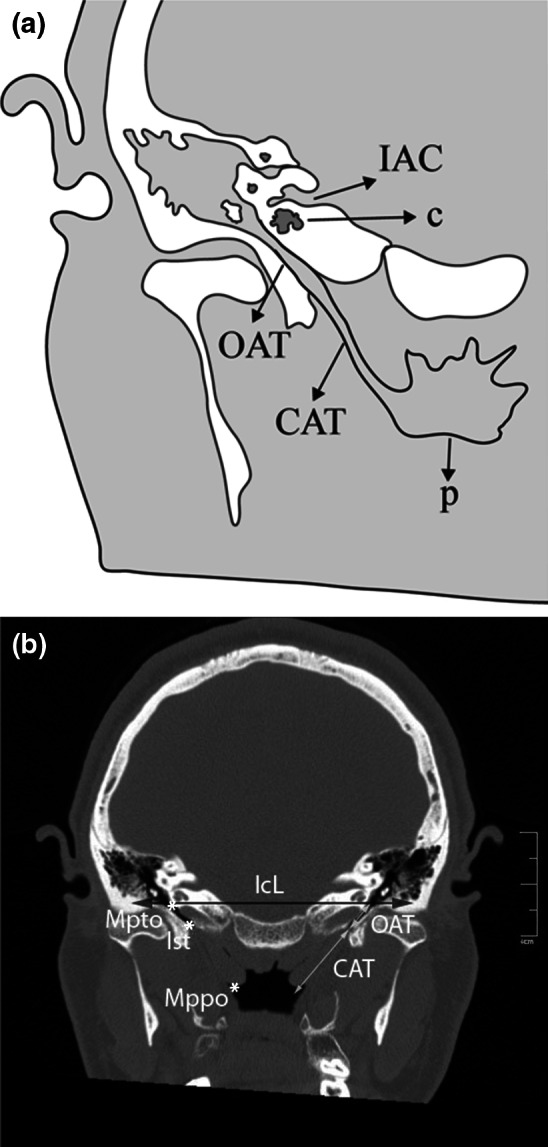
**a** Oblique coronal schematic illustration showing the auditory tube and related structures. **b** Reformatted oblique coronal computed tomography image showing *reference points* and *lines*. *CAT* cartilaginous auditory tube length, *c* cochlea, *IAC* internal acoustic canal, *IcL* intercochlear line, *Ist* isthmus, *Mppo* midpoint of the pharyngeal opening of the auditory tube, *Mpto* midpoint of the tympanic opening of the auditory tube, *OAT* osseous auditory tube length, *p* pharynx

**Fig. 3:**
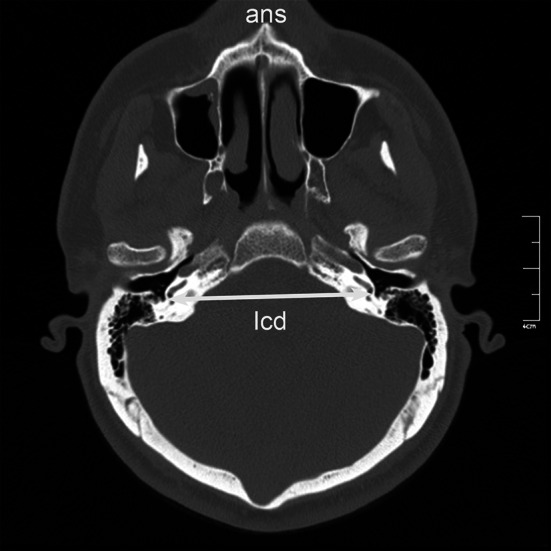
Reformatted computed tomography image showing intercochlear distance (*IcD*) and anterior nasal spine (*ans*)

**Fig. 4:**
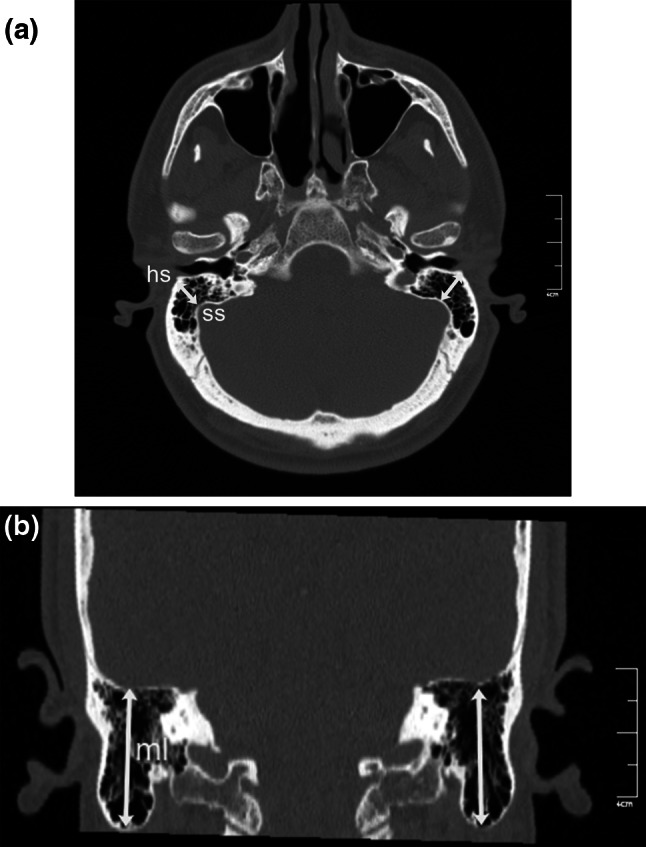
Reformatted computed tomography images. **a** Oblique axial image showing Henle suprameatal spine (*hs*) to sigmoid sinus (*ss*) distance (mastoid depth). **b** Coronal image showing mastoid length (*ml*) which was the shortest distance between the mastoid tip and tegmen tympani

**Fig. 5:**
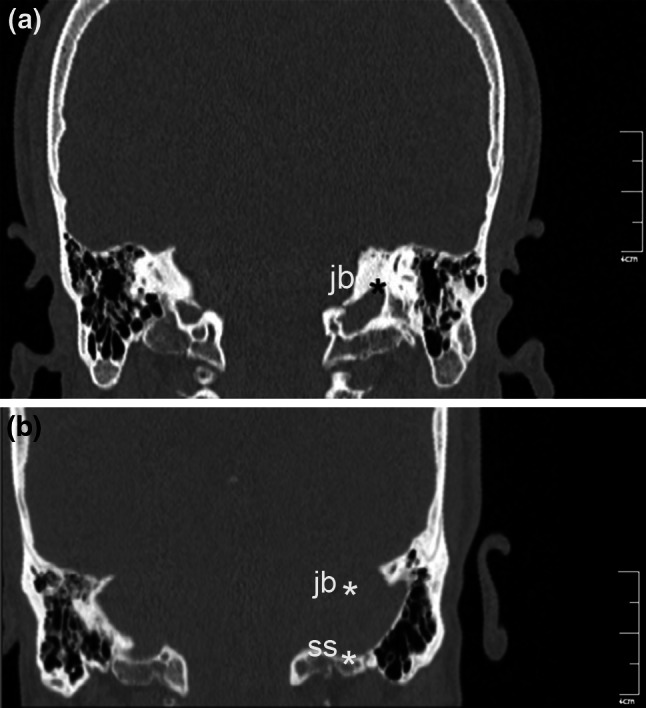
Reformatted coronal computed tomography images. **a** The *cursor* is on the highest point of the jugular bulb. **b** The deepest point of the sigmoid sinus (*ss*) is noted, and the height of the jugular bulb (*ss-jb*) is the distance between level of the jugular bulb dome and a *line* passing through confluence of the sigmoid sinus (*ss*) with jugular bulb

**Fig. 6:**
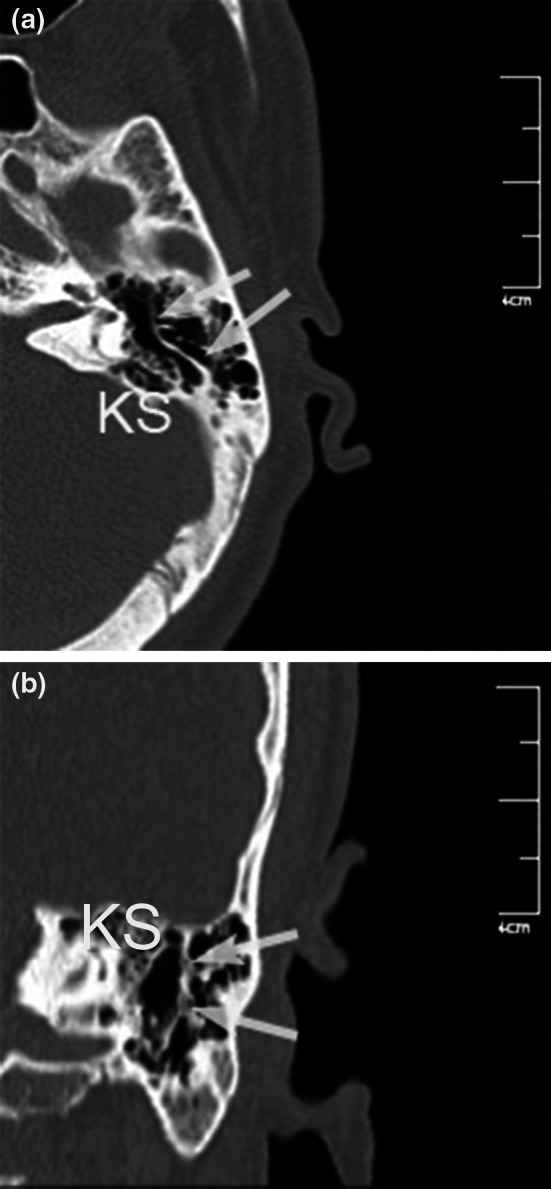
Reformatted computed tomography images. **a** Oblique axial image. **b** Coronal image. The Körner (petrosquamosal) septum (*KS*) is noted

**Fig. 7:**
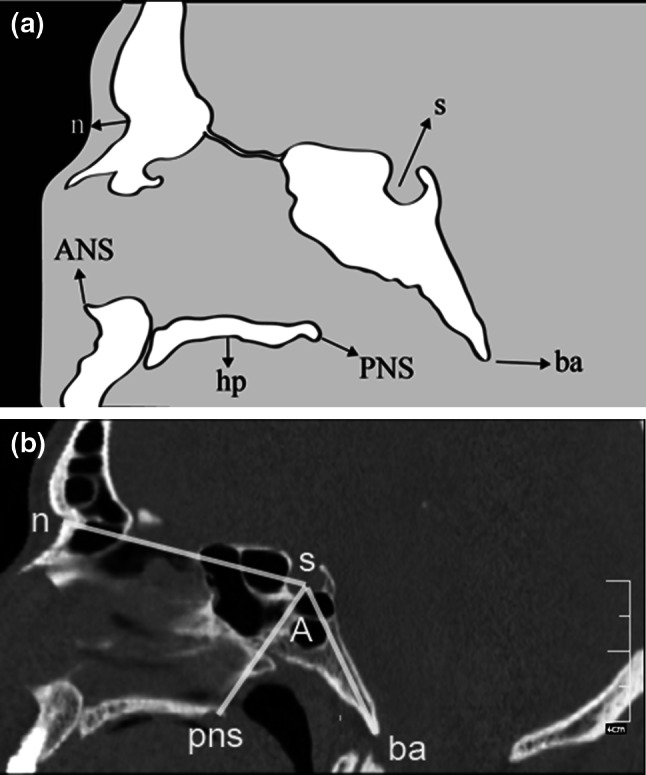
**a** Schematic representation of the midsagittal structures and landmarks. **b** Reformatted midsagittal computed tomography image. *A* angle (measured at center of sella turcica) between sella-basion line and line through center of sella turcica and posterior nasal spine. *ANS* anterior nasal spine, *ba* basion, *hp* hard palate, *PNS* posterior nasal spine, *n* nasion, *s* center of sella turcica

**Fig. 8:**
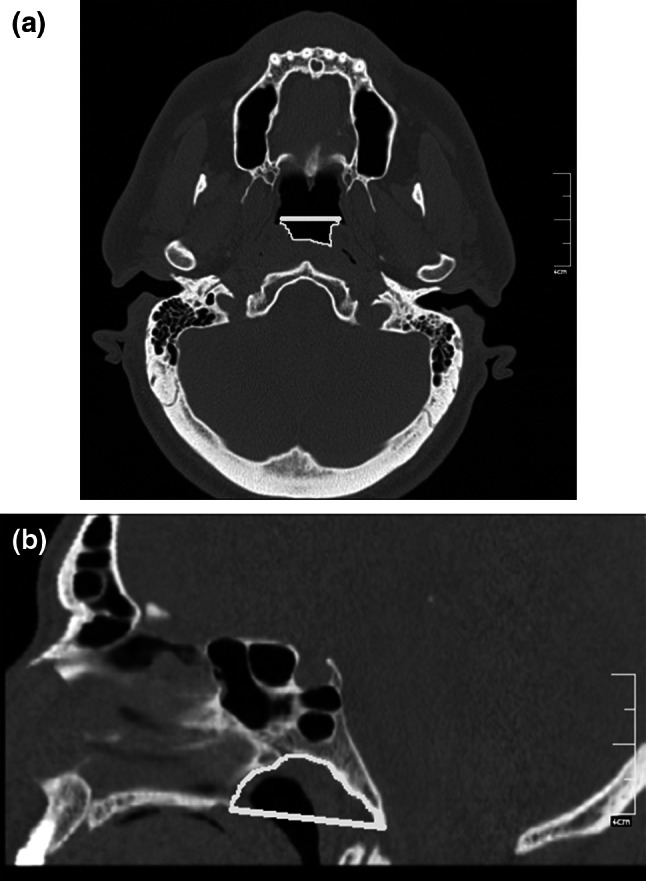
Reformatted computed tomography images. **a** Oblique axial image showing nasopharynx axial area (*Npax*) and the distance between the midpoints of the pharyngeal openings (*Mppod*). **b** Midsagittal image showing nasopharynx sagittal area (*Npsag*)

The axial CT image was rotated coronally, and oblique coronal reformatted CT images were obtained. To solve the asymmetry problem arising from patient position, the angle of the reformatted image was adjusted until basal turns of both cochleae became visible. The images were standardized for all patients; reference points and lines were defined, and multiple measurements (linear, angular, and area measurements) were made from each CT scan (Table 1; Figs. 1, 2, 3, 4, 5, 6, 7, 8). Reformatted images were recorded on a workstation program, and measurements were done electronically. Measurements were performed twice at different sessions for each patient, and arithmetic means were calculated to minimize random erroneous measurements.

**Table 1:** Definitions of reference points, reference lines, and parameters from craniofacial computed tomography scans

Category		
Abbreviation	Item	Definition
Reference points		
ans	Anterior nasal spine	Midpoint of anterior edge of nasal spine
pns	Posterior nasal spine	Midpoint of posterior edge of hard palate (staphylion)
ba	Basion	Most posteroinferior point of spheno-occipital bone on anterior margin of foramen magnum
hs	Henle suprameatal spine	Henle suprameatal spine
n	Nasion	Most anterior point of frontonasal suture
s	Sella	Center of sella turcica
ss	Sigmoid sinus	Sigmoid sinus
Mpto	Midpoint of tympanic opening of auditory tube	Midpoint of tympanic opening of auditory tube
Ist	Isthmus	Most distal visible end of the bony auditory tube
Mppo	Midpoint of pharyngeal opening of auditory tube	Point nearest pharynx where a loop-shaped auditory tube lumen appears
Reference lines		
NSL	Nasion-sella line	Line through nasion and sella
SbaL	Sella-basion line (posterior cranial base line)	Line through sella and basion
IcL	Intercochlear line	Line through cochleae
OATL	Osseous auditory tube line	Line through “Mpto” and isthmus
Linear parameters		
OAT	Osseous auditory tube length	Distance from “Mpto” to isthmus
CAT	Cartilaginous auditory tube length	Distance from isthmus to the “Mppo”
IcD	Intercochlear distance	Distance between most lateral points of basal turns of cochleae
s-n	Anterior cranial base-ACB-length	Distance from sella to nasion
s-pns	Posterior upper face height (PUFH)	Distance from sella to posterior nasal spine
s-ba	Posterior cranial base (PCB) length	Distance from sella to basion
hs-ss	Mastoid depth	Shortest distance between base of hs (lateral border) and sigmoid sinus
ml	Mastoid length	Shortest distance between mastoid tip and tegmen tympani
ss-jb	Height of the jugular bulb	Distance between level of jugular bulb dome and line passing through confluence of the sigmoid sinus with jugular bulb
KS	Körner (petrosquamosal) septum	Thickness of septum measured at thickest point on coronal image
Mppod		Distance between the midpoints of the pharyngeal openings
Angular parameters		
AAT	Angle of the auditory tube	Angle between IcL and OATL
CBA	Cranial base angle	Angle between NSL and SbaL (ba.s.n)
A	Angle ba.s.pns	Angle (measured at sella) between SbaL and line through sella and posterior nasal spine
Area parameters		
Npax	Nasopharynx axial area	Area between midpoints of pharyngeal openings of auditory tube (Mppo) and posterior border of pharyngeal soft tissue
Npsag	Nasopharynx sagittal area	Area between posterior border of vomer, inferior border of sphenoid sinus, anterior border of clivus, and basion to posterior nasal spine line

The distance between midpoint of the tympanic openings of the auditory tube and the most distal visible end of the bony auditory tube (isthmus) was defined as the osseous auditory tube length (Table 1; Fig. 2). The distance between the isthmus and the midpoint of the pharyngeal openings of the auditory tube was defined as the cartilaginous auditory tube length (Table 1; Fig. 2). For measurement of the intercochlear distance, which was the breadth of the cranial base, the cursor was placed on the anterior nasal spine; the image was rotated coronally until both basal turns came into sight; and the intercochlear distance was measured as the distance between the most lateral points of the basal turns of the cochleae (Table 1; Fig. 3).

Mastoid depth was defined as the distance between Henle suprameatal spine and the sigmoid sinus (Table 1; Fig. 4). The cursor was placed in the middle of the line joining right and left Henle spines to obtain an oblique axial image in which both the longest lengths of both Henle spines were observed. The shortest distance between the base of Henle spine and the anterior edge of the sigmoid sinus was measured as mastoid depth (Fig. 4). To measure the mastoid length, the angle of the reformatted coronal image was adjusted until the most inferior point of both mastoid tips came into view; images were standardized for all patients, and the mastoid length was measured as the shortest distance between the mastoid tip and the tegmen tympani (Table 1; Fig. 4).

Height of the jugular bulb was the distance between the level of the jugular bulb dome and the line passing through the confluence of the sigmoid sinus. The cursor was placed on the highest point of the jugular bulb on the coronal image (Fig. 5); sections were followed posteriorly until the deepest point of the sigmoid sinus came into view, and the distance between the cursor and the sigmoid sinus was measured (Table 1; Fig. 5). The presence and thickness of Körner (petrosquamosal) septum were assessed on both axial and coronal images (Table 1; Fig. 6). Cephalometric measurements were obtained on the reformatted midsagittal image (Fig. 7). Nasopharynx axial area and distance between midpoints of pharyngeal openings were measured from the oblique axial images, and the nasopharynx sagittal area was measured on the midsagittal images (Table 1; Fig. 8).

### Statistical analyses

Data analysis was performed with statistical software (IBM SPSS for Windows, Version 21.0, IBM Corp, Armonk, NY, USA). Continuous variables were reported as mean ± standard deviation. Normality of the continuous variables was evaluated with Shapiro–Wilk test. Differences in continuous variables between two groups were evaluated with *t* test for independent samples. Comparison of mastoid size and jugular bulb height for ears that had presence or absence of Körner septum was performed with Mann–Whitney test. Relation between continuous variables was evaluated with Pearson product moment correlation. Statistical significance was defined by *P* ≤ .05.

## Results

Compared with women, men had significantly greater mean osseous auditory tube length, cartilaginous auditory tube length, mastoid length, intercochlear distance, sella to posterior nasal spine distance, sella to basion distance, and nasopharynx sagittal area (Table 2). There were no differences in the other parameters between men and women (Table 2).

**Table 2:** Craniofacial parameters in adults determined from two-dimensional computed tomography scans

Parameter type	Parameter	Side	Abbreviation	Men	Women	*P* ≤^a^
No. of patients				30	30	
Aged (years)				37 ± 12	39 ± 12	NS
Bilateral craniofacial antimeric						
Osseous auditory tube length (mm)		Right	OATR	13 ± 1	12 ± 1	.003
Osseous auditory tube length		Left	OATL	13 ± 1	12 ± 1	.01
Cartilaginous auditory tube length (mm)		Right	CATR	30 ± 2	29 ± 2	.05
Cartilaginous auditory tube length		Left	CATL	30 ± 2	29 ± 2	.01
Mastoid depth (Henle suprameatal spine to sigmoid sinus distance) (mm)		Right	hs-ssR	15 ± 3	15 ± 3	NS
Mastoid depth (Henle suprameatal spine to sigmoid sinus distance)		Left	hs-ssL	15 ± 3	15 ± 3	NS
Mastoid length (mm)		Right	mlR	42 ± 4	39 ± 3	.001
Mastoid length		Left	mlL	42 ± 5	38 ± 3	.001
Jugular bulb height (mm)		Right	ss-jbR	13 ± 3	12 ± 4	NS
Jugular bulb height		Left	ss-jbL	12 ± 5	11 ± 4	NS
Angle of the auditory tube		Right	AATR	44 ± 5	45 ± 4	NS
Angle of the auditory tube		Left	AATL	46 ± 4	47 ± 4	NS
Other						
Intercochlear distance (mm)			IcD	78 ± 4	74 ± 4	.001
Midpoints of the pharyngeal opening distance (mm)			mppod	22 ± 3	21 ± 2	NS
Sella to nasion distance (mm)			s-n	61 ± 5	59 ± 4	NS
Sella to posterior nasal spine distance (posterior upper face height) (mm)			s-pns (PUFH)	47 ± 4	45 ± 3	.02
Sella to basion distance (mm)			s-ba	45 ± 3	44 ± 3	.04
Nasopharynx axial area (mm^2^)			npax	181 ± 55	177 ± 46	NS
Nasopharynx sagittal area (mm^2^)			npsag	498 ± 81	424 ± 53	.001
Cranial base angle			CBA	125 ± 81	123 ± 6	NS
Angle at sella between s-ba and s-pns^b^			A	50 ± 5	48 ± 4	NS

*N* = 60 patients. Data reported as mean ± SD

^a^ *NS* not significant (*P* > .05)

^b^Angle at sella between sella to basion distance line and line through sella and posterior nasal spine

The intercochlear distance was significantly correlated with mastoid depth, midpoint of the pharyngeal opening distance, sella to nasion distance, and nasopharynx sagittal area and inversely with angle of the auditory tube (Table 3). Most men and women had Körner septum present, and mean thickness of Körner septum was significantly greater in men than women (Table 4). Mean mastoid depth, mastoid length, and jugular bulb height were similar in patients who had Körner septum present or absent (Table 4).

**Table 3:** Relation between intercochlear distance and other craniofacial parameters

Parameter	Side	Abbreviation	*r*	*P* ≤^a^
Osseous auditory tube length	Right	OATR	0.157	NS
Osseous auditory tube length	Left	OATL	0.23	NS
Cartilaginous auditory tube length	Right	CATR	0.155	NS
Cartilaginous auditory tube length	Left	CATL	0.1	NS
Mastoid depth (Henle suprameatal spine to sigmoid sinus distance)	Right	hs-ssR	0.305	.02
Mastoid depth (Henle suprameatal spine to sigmoid sinus distance)	Left	hs-ssL	0.319	.02
Mastoid length	Right	mlR	0.138	NS
Mastoid length	Left	mlL	0.209	NS
Jugular bulb height	Right	ss-jbR	0.145	NS
Jugular bulb height	Left	ss-jbL	0.115	NS
Angle of the auditory tube	Right	AATR	−0.263	.05
Angle of the auditory tube	Left	AATL	−0.441	.001
Midpoint of the pharyngeal opening distance		mppod	0.491	.001
Sella to nasion distance		s-n	0.283	.03
Sella to posterior nasal spine distance (posterior upper face height)		s-pns (PUFH)	0.223	NS
Sella to basion distance		s-ba	0.146	NS
Nasopharynx axial area		npax	0.243	NS
Nasopharynx sagittal area		npsag	0.318	.02
Cranial base angle		CBA	0.092	NS
Angle at sella between s-ba and s-pns^b^		A	0.035	NS
Körner (petrosquamosal) septum	Right	KSR	0	NS
Körner (petrosquamosal) septum	Left	KSL	0.187	NS

*r* correlation coefficient

^a^ *NS* not significant (*P* > .05)

^b^Angle at sella between sella to basion distance line and line through sella and posterior nasal spine

**Table 4:** Comparison of craniofacial parameters for ears with and without Körner (petrosquamosal) septum

Sex	Parameter	Abbreviation	Körner (petrosquamosal) septum^a^		*P* ≤^b^
			Present	Absent	
Men					
No. of patients			45 (75)	15 (25)	
Mastoid depth (Henle suprameatal spine to sigmoid sinus distance)		hs-ss	15 ± 3	15 ± 3	NS
Mastoid length		ml	43 ± 5	41 ± 4	NS
Jugular bulb height		ss-jb	13 ± 4	12 ± 4	NS
Women					
No. of patients			38 (63)	22 (37)	
Mastoid depth (Henle suprameatal spine to sigmoid sinus distance)		hs-ss	15 ± 3	14 ± 3	NS
Mastoid length		ml	38 ± 4	39 ± 3	NS
Jugular bulb height		ss-jb	11 ± 4	11 ± 4	NS

*N* = 30 men and 30 women. Data reported as number (%) or mean ± SD

^a^Körner (petrosquamosal) septum thickness: men 1.0 ± 0.2 mm; women 0.9 ± 0.2 mm; *P* ≤ .001

^b^ *NS* not significant (*P* > .05)

## Discussion

In the present study, craniofacial measurements in a homogenous population of Turkish people who had no otitis media showed that several mean parameters were greater in men than women (Table 2). In addition, the intercochlear distance (breadth of cranial base) was significantly correlated with several craniofacial parameters (Table 3). Reformatted two-dimensional CT scanning may enable measurement of midsagittal parameters and bilateral craniofacial antimeric parameters in living subjects. However, CT scanning requires more radiation than cephalometry. In addition, the reformatting process in CT scanning is limited by the slice thickness, and error may be expected.

The total nasopharyngeal depth is established in the first or second year of life, but increase in nasopharyngeal height continues until maturity. Restriction of the nasopharyngeal airway may occur during the preschool and early school years because of adenoid hypertrophy that may exceed the typical increase in nasopharyngeal capacity [9]. Children who have otitis media with effusion have a smaller nasopharynx than other children, possibly because of a difference in the rate and timing of growth [12]. The maximum nasopharyngeal capacity may be achieved by 13.75 years in females and 17 and 18 years in males. Growth of the nasopharynx parallels the sexually determined growth of the skeleton [9]. The craniofacial skeleton develops postnatally up to age of 19 years, and all dimensions of the temporal bone and dimensions related to the ventral surface of the sphenoid bone increase until this age [2, 11, 14, 18]. Therefore, we included nongrowing adults (age > 19 years) to avoid age-related changes in the data. The present study showed significantly greater nasopharyngeal sagittal area in male than female patients, but the nasopharyngeal axial area and midpoint of the pharyngeal opening distance were similar between adult male and female subjects (Table 2). Sella to nasion distance, midpoint of the pharyngeal opening distance, and nasopharynx sagittal area correlated with IcD (Table 3).

In patients aged 10–15 years (but not other age groups), girls have a larger mastoid air cell system than boys; this sex difference may be attributed to the hormonally induced growth of the cellular system, which is faster in girls immediately before and during puberty [24]. In addition, mastoid length (porion to mastoid process length) has significant sex difference, and growth of this dimension is strongly related to physical and neural growth and development of the human head [21]. Before puberty, the mastoid system usually matures and pneumatization increases; many atelectatic conditions are halted or reversed, and the middle ear may revert to normal [16]. Mastoid length is shorter in poorly pneumatized mastoids, but there is no correlation between lateral sinus and external auditory canal distance and the degree of pneumatization; however, this information was based on a study that was performed on unselected, fresh frozen, adult temporal bones and there was no information regarding sex [25]. In our previous study, we observed statistically significant differences in mastoid length and depth between patients who had chronic otitis media and healthy sex- and age-matched adults [1]. In the present study, mastoid length was longer in men than women, but there was no difference in mastoid depth between male and female subjects (Table 2). The IcD was correlated with mastoid depth but not mastoid length (Table 3).

The auditory tube is almost flat in children and progressively rotates inferomedially to assume its final position during puberty [19]. Otitis media is most common during childhood, when tubal dysfunction is more prevalent. The decrease in the incidence of otitis media with maturity has been correlated with the shift in the position of the auditory tube, which is more vertical in adults. The change in auditory tube position is caused by growth of the craniofacial skeleton [5]. Increased length of the auditory tube is associated with greater degree of pneumatization of the mastoid air cell system, suggesting an association between nasopharyngeal morphology and the development of otitis media. In addition, healthy middle ears in adults typically have long auditory tubes, long distances from the midsella to the staphylion, and longer distances between the ears [23]. The latter finding is inconsistent with reports that brachycephalic people have a higher frequency of otitis media than dolichocephalic people [20]. A recent study did not support a difference in the cephalic index among children with recurrent AOM, chronic otitis media with effusion, and control subjects in which the statistical methods included provisions to control for race, sex, and age [3]. In the present study, osseous auditory tube length, cartilaginous auditory tube length, posterior upper face height (sella to posterior nasal spine distance), sella to basion distance, and Icd differed significantly between men and women (Table 2). In addition, a significant negative correlation was observed between Icd and angle of the auditory tube (Table 3).

There is controversy about the relation between pneumatization of the mastoid and high jugular bulb [8, 15]. In the present study, no differences were observed between men and women in height of the jugular bulb (Table 2).

Körner (petrosquamosal) septum may have clinical importance because it is associated with some ear problems [7]. In addition, smaller air cell systems may be associated with larger septa [17]. In the present study, the prevalence and mean thickness of Körner septum were greater in men than women (Table 4). In addition, no differences were observed when mastoid depth, mastoid length, and jugular bulb height were compared between ears that had or did not have Körner septum (Table 4).

In summary, the present study showed that osseous auditory tube length, cartilaginous auditory tube length, mastoid length, posterior upper face height, sella to basion distance, nasopharynx sagittal area, and IcD differed significantly between men and women (Table 2). The greatest skeletal differences between men and women were observed in the vertical craniofacial dimensions. The Icd positively correlated with mastoid depth, midpoint of the pharyngeal opening distance, sella to nasion distance, and nasopharynx sagittal area but negatively correlated with angle of the auditory tube (Table 3). The prevalence and thickness of the Körner septum were different between men and women (Table 4). Therefore, some craniofacial parameters, especially vertical parameters, differ with sex. These differences begin in childhood and continue in adulthood. Therefore, sex must be considered when planning a craniofacial morphologic study, and results of a craniofacial morphologic study should be evaluated with caution when there is no sex matching of the patient and control groups.
